# AHP-express: A simplified version of the analytical hierarchy process method

**DOI:** 10.1016/j.mex.2019.11.021

**Published:** 2019-12-04

**Authors:** José Eugenio Leal

**Affiliations:** Pontifical Catholic University of Rio de Janeiro, Brazil

**Keywords:** AHP-express, AHP, Multicriteria decision making, Business application

## Abstract

This work presents in detail a simplified method for the application of the analytic hierarchy method (AHP) that aims to calculate the priorities of each alternative against a set of criteria with only n-1 comparisons of n alternatives for each criterion (instead of (n^2^-n)/2 comparisons in the original method proposed by Saaty), followed by the application of a simple formula. This increases the attractiveness of the AHP method for business applications where decisionmakers are subject to a time constraint.

• Simplified AHP method.
• Reduced number of comparisons.
• Saved time of highly qualified technical staff.

## Graphical abstract

**Figure:**
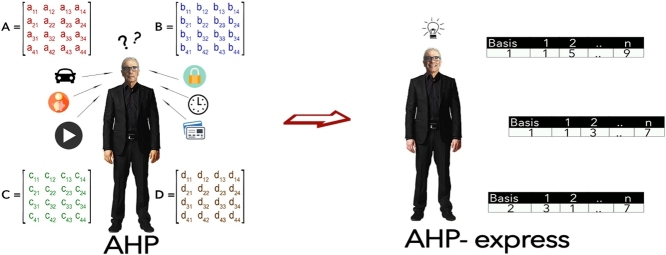

## Specification Table

**Table:** 

**Subject area:**	Engineering
**More specific subject area:**	Decision Making Science. Industrial Engineering
**Method name:**	AHP-express
**Name and reference of original method:**	AHP
**Resource availability:**	Excel spreadsheet

## Method details

### Background

The analytic hierarchy method (AHP), developed by Saaty, is a powerful multicriteria decision-making tool that has been used in numerous applications in various fields of economics, politics and engineering.

According to Chai et al. [1], the AHP method makes it possible to assign a value representing the preference degree for a given alternative to each additional alternative. Such values can be used to classify and select alternatives based on a hierarchical structure. Gupta et al. [2] and Jadhav and Sonar [3] noted that AHP is the most widely used method for evaluating software. AHP has also been applied to supplier and vendor selection according to Tam and Tummala [4], Deng et al. [5], and Rajesh and Malliga [6]. Recent approaches have combined AHP with other methods as presented by Ahn [7]. Mendes et al. [11] and Guimarães et al. [12] have applied this new AHP approach in their work. Interested readers can find a brief discussion on AHP applications in the supplementary material.

Despite its wide applicability, the AHP method suffers from a notable drawback: it requires a very large number of comparisons to make a decision. This condition hampers its application to the most important issues, which involve the participation of senior executives in organizations. Therefore, assuming evaluation consistency by decisionmakers, a method that reduces the number of comparisons for each criterion or between criteria is proposed, in which comparisons are made only between one element and all other elements. It is suggested that the element taken as a basis is one of apparently greater importance—one whose inconsistency of evaluation would be expected to be the least likely in an application of the complete method.

The method starts by structuring a decision-making problem as a hierarchy in the form of an upside-down tree where the main goal is placed on top. Partial objectives that meet the main objective are placed at the second level. Each partial objective at the second level can be decomposed into third-level objectives, and each set at each level meets the objective of the level to which they are subordinate. These partial objectives are treated as criteria in this text. At a lower level, the alternatives are listed and then compared pairwise according to their contribution to reaching each objective, or criterion, from the lower level. Pairwise comparisons are performed using the method described by Saaty [8] at the fundamental scale presented in Table 1.

**Table 1:** Fundamental scale of Saaty.

Intensity of importance	Definition
1	Equal importance
3	Moderate importance
5	Strong importance
7	Very strong importance
9	Extreme importance

An ***nXn*** matrix is assembled, where ***n*** is the number of alternatives. Considering a given criterion, matrix ***A*** is supplemented with values ***a_ij_***, where ***i*** is a base alternative for comparison, corresponding to row ***i***, and ***j*** is the alternative being compared with ***i***. If the contribution of ***i*** to the criterion being considered is of strong importance relative to ***j***, ***a_ij_*** assumes the value of 5, which can be considered a dominance of ***i*** over ***j***. Intermediate values between those shown can also be considered. The procedure presents some important relationships in the matrix:

(1) aji=1aij

In the case of consistent evaluations:

(2) ajk=aikaij

where ***k*** and ***j*** are two alternatives being compared to ***i***.

After the matrix is complete, the process searches for a vector that expresses the priority of each alternative for the considered criterion. For Saaty, this vector of priorities ***x*** is obtained by starting from the relation between matrix ***A***, its greater eigenvalue ***λ*** and the corresponding vector ***x***.

(3) ***Ax= λx***.

Comparisons are made between each alternative against each criterion and between each criterion at one level against the higher-level criterion to which it is associated. Finally, each criterion of the first level is compared against the objective. The comparison is made by assembling matrices in the same way and with the same scale as presented in Table 1. The priorities of the criteria are used as weights to calculate the priorities of the alternatives in each criterion until the priorities of the alternatives against the overall objective have been calculated.

According to the method proposed by Saaty, given relation 1 and the fact that the diagonal ***a_ij_*** = 1, ***(n*^2^*-n)/2*** comparisons are made before proceeding with the calculations of priorities for each matrix with n alternatives.

When making multiple comparisons, inconsistencies typically occur that are acceptable within certain limits. Consequently, Saaty defined how to compute an inconsistency indicator from an array of evaluations and described a test with a parameter to check whether evaluation inconsistency is acceptable. Appendix A1 of the supplementary material shows approximate methods for estimating the vector of priorities and how to test the inconsistency of the evaluation matrix. The method has been applied and validated in the literature as presented by Mendes et al. [11] and Guimarães et al. [12] and in practical situations as reported by do Nascimento Vieira and Leal [9] and Nunes and Leal [10].

#### Steps of the AHP-express method

The method is based on the assumption of evaluation consistency; it also relies on the hypothesis that inconsistency occurs mainly in evaluations between alternatives of seemingly minor importance to the decisionmaker. By taking as its basis alternatives of apparent greater importance, the method makes a more careful analysis when comparing these alternatives with the other ones. Thus, it is proposed that for each calculation of priorities in each criterion, the alternative that the decisionmaker considers the most or one of the most important alternatives is taken as the basis of comparison. Thereafter, the comparison is made using the evaluation scale proposed by Saaty between this alternative and all other alternatives, and then, the elements of the vector of priorities can be calculated by means of formula (4).

(4) prj=1aij*∑k1aik 

where ***j*** is the element for which one wishes to calculate the priority, ***i*** is the element taken as the basis for the comparison, ***a_ij_*** is the comparison value of alternative ***i*** with alternative ***j***, and ***pr_j_*** is the priority of alternative ***j*** against the criterion considered. The development of this formula is found in the supplementary material along with a proof that this formula gives the elements of the eigenvector associated with the main eigenvalue, in the case of consistency in the matrix.

The following are the steps for the simplified method.

1 Set the main purpose of the decision-making process.
2 Define the secondary objectives that together meet the primary objective at the second level of the structure of priorities.
3 For each objective at the second level, if necessary, define third-level objectives that meet the next higher objective.
4 Define the alternatives to be considered at the lower level.
5 For each element in a level, repeat the following:
  a Set the element of apparent greater importance with respect to the criterion of the higher level.
  b Apply formula 4 to calculate the elements of the vector of priorities for the criterion under consideration.
6 Calculate the priorities of each alternative within each criterion ascending in the tree to the main objective.

This method can also be applied for the pairwise comparisons used in the analytical network process (ANP, [8]).

The procedure can be represented in matrix form.

Define:

a: alternative a, a = 1,…,na

Ci: criterion i, i = 1,…,nc

SCi,j: subcriterion j within criterion i, j = 1,…,nsi

For each criterion i, we calculate the following:

A vector PSCi of priorities of subcriterion j in criterion i:

(6) $${PSC}_{i}=\left[\begin{array}{ccc} {cg}_{1}^{i} & \ldots & {cg}_{nsi}^{i} \end{array}\right]$$

A matrix PASC i of priorities of alternative a in each subcriterion j of criterion i:

(7) $${PASC}_{i}=\left[\begin{array}{ccc} {pasc}_{1,1}^{i} & .. & {pasc}_{na,1}^{i}\\ . & . & .\\ {pasc}_{1,{ns}_{i}}^{i} & . & {pasc}_{na,ns{\;}_{i}}^{i} \end{array}\right]$$

Each matrix PASCi can be ordered by each criterion i grouped in a matrix MPASC:

(8) $$MPASC=\left[\begin{array}{ccc} {pasc}_{1,1}^{i} & \ldots & {pasc}_{na,1}^{i}\\ \ldots & \ldots & \ldots \\ {pasc}_{1,{ns}_{i}}^{i} & \ldots & {pasc}_{na,ns{\;}_{i}}^{i}\\ \ldots & \ldots & \ldots \\ {pasc}_{1,1}^{nc} & & {pasc}_{na,1}^{nc}\\ \ldots & \ldots & \ldots \\ {pasc}_{1,nsnc}^{nc} & & {pasc}_{na,nsnc}^{nc} \end{array}\right]$$

Each vector PSCi can be included in a matrix MPSC of priorities of subcriteria, which is partitioned to correspond to the priorities of alternatives in matrix MPASC:

(9) $$\text{M}\text{P}\text{A}\text{S}\text{C}=\left[\begin{array}{cccccccc} {cg}_{1}^{1} & & {cg}_{ns1}^{1} & 0 & .. & 0 & 0 & 0\\ 0 & 0 & 0 & {cg}_{1}^{2} & .. & {cg}_{ns2}^{2} & 0 & 0\\ .. & .. & .. & .. & .. & .. & .. & ..\\ 0 & 0 & 0 & 0 & 0 & 0 & {cg}_{1}^{nc} & {cg}_{nsnc}^{nc} \end{array}\right]$$

The product of MPSC by PASC gives the matrix PAC of priorities for each alternative a for criteria i. Each row of matrix PAC correspond to a vector of priorities of alternatives in each criteria i.

(10) $$MPSC\text{*}PASC=PAC=\left[\begin{array}{ccc} {pac}_{1,1} & .. & {pac}_{na,1}\\ .. & .. & ..\\ {pac}_{1,nc} & .. & {pac}_{na,nc} \end{array}\right]$$

Further, we calculate a vector PC of priorities of each criterion for the objective.

(11) $$PC=\left[\begin{array}{ccc} {pc}_{1} & .. & {pc}_{nc} \end{array}\right]$$

The product of PC and PAC gives the vector PA of final priorities for each alternative for the objective.

(12) $$PA=PC\text{*}PAC=\left[\begin{array}{ccc} p_{1} & \ldots & p_{na} \end{array}\right]$$

**Fig. 1:**
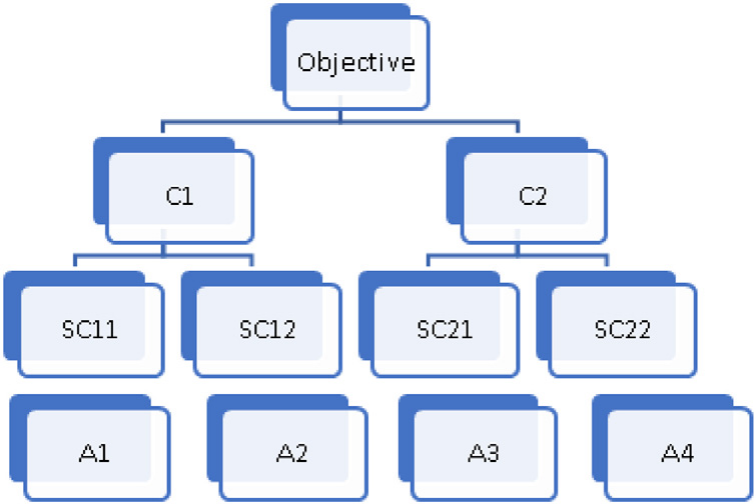
Example of objective hierarchy with two levels of criteria and alternatives in the third level.

**Fig. 2:**
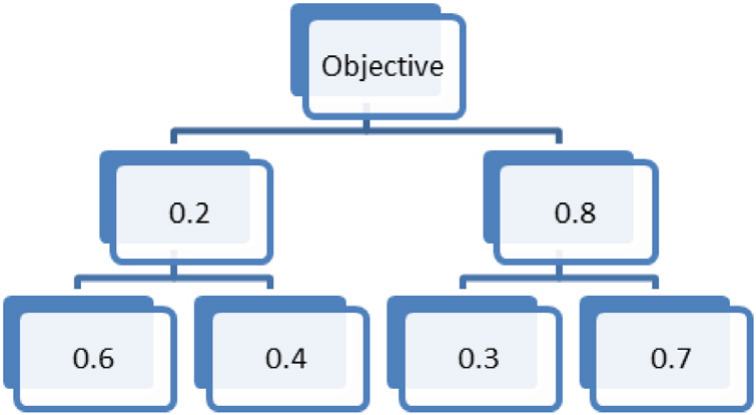
Example of subcriteria priorities within each criterion and criteria against the objective.

Example:

Four alternatives with two subcriteria for each of two criteria (Fig. 1, Fig. 2).

In our example, we have four alternatives (***A1, A2, A3*** and ***A4***), two criteria (***C1*** and ***C2***) and two subcriteria for each criterion (***C11***, ***C12***, ***C21*** and ***C22***).

The calculation of the priorities for the alternatives of each subcriteria, using Formula 4, are presented in Table 2, Table 3, Table 4, Table 5.

**Table 2:** Priority calculation for subcriteria SC11.

**Table 3:** Priority calculation for subcriteria SC12.

**Table 4:** Priority calculation for subcriteria SC21.

**Table 5:** Priority calculation for subcriteria SC22.

For each subcriterion, we can group the priorities of the alternatives in a matrix:

$${PASC}_{i}=\left[\begin{array}{ccc} {pasc}_{1,1}^{i} & .. & {pasc}_{na,1}^{i}\\ . & . & .\\ {pasc}_{1,{ns}_{i}}^{i} & . & {pasc}_{na,ns{\;}_{i}}^{i} \end{array}\right]$$

For subcriteria ***C11*** and ***C12*** of criterion ***1,*** we have:

Each matrix ***PASCi*** can be ordered by each criterion ***i*** grouped in a matrix ***PASC***:

$$PASC=\left[\begin{array}{ccc} {pasc}_{1,1}^{i} & \ldots & {pasc}_{na,1}^{i}\\ \ldots & \ldots & \ldots \\ {pasc}_{1,{ns}_{i}}^{i} & \ldots & {pasc}_{na,ns{\;}_{i}}^{i}\\ \ldots & \ldots & \ldots \\ {pasc}_{1,1}^{nc} & & {pasc}_{na,1}^{nc}\\ \ldots & \ldots & \ldots \\ {pasc}_{1,nsnc}^{nc} & & {pasc}_{na,nsnc}^{nc} \end{array}\right]$$

A vector (***PSCi***) of the priorities of subcriterion j in criterion i is calculated for each criterion (Table 6, Table 7):

$${PSC}_{i}=\left[\begin{array}{ccc} {cg}_{1}^{i} & \ldots & {cg}_{nsi}^{i} \end{array}\right]$$

**Table 6:** Matrix of priorities of the alternatives in the subcriteria of criterion 1.

**Table 7:** Matrix of the priorities of the alternatives in each subcriterion (PASC).

The calculation of the priorities for each subcriterion in each criterion is presented in Table 8, Table 9.

**Table 8:** Calculation of the priorities of the subcriteria of criterion 1.

**Table 9:** Calculation of the priorities of the subcriteria of criterion 2.

The priorities are grouped in a matrix ***PSC***, which is partitioned to multiply the weights of the subcriteria in the corresponding criteria by the priorities of the alternatives in the corresponding subcriteria (Table 10).

**Table 10:** Matrix of the priorities of the subcriteria for each criterion (PSC).

The product of multiplying ***PSC*** by ***PASC*** (see the schema in Table 11) is the matrix ***PAC*** (Table 12), which contains the priorities for each alternative ***a*** for criterion ***i***. Each row of the ***PAC*** matrix (Table 13) corresponds to a vector of the priorities of the alternatives for each criterion ***i***.

$$PSC\text{*}PASC=PAC=\left[\begin{array}{ccc} {pac}_{1,1} & .. & {pac}_{na,1}\\ .. & .. & ..\\ {pac}_{1,nc} & .. & {pac}_{na,nc} \end{array}\right]$$

**Table 11:** Schema of the matrix multiplication PSC*PASC.

**Table 12:** Priorities of the alternatives in each criterion (PAC).

**Table 13:** Calculation of priorities of each criterion.

The result of this matrix multiplication is the matrix ***PAC***, which will be multiplied by the vector ***PC*** of the priorities of the criteria as shown in Table 13:

The final priorities of the alternatives ***PA*** (Table 15) are calculated by the matrix multiplication PC*PAC following the schema shown in Table 14.

**Table 14:** Schema of the matrix calculation of the final priorities.

**Table 15:** Vector (PA) of the final priorities of the alternatives.

The diagram of Fig. 4 resumes the steps of the method for a hierarchy with criteria, subcriteria and alternatives. The box at the left calculates priorities for a given set of ***n_e_*** elements that can be alternatives, subcriteria or criteria with respect to an upper-level element in the hierarchy.

**Fig. 3:**
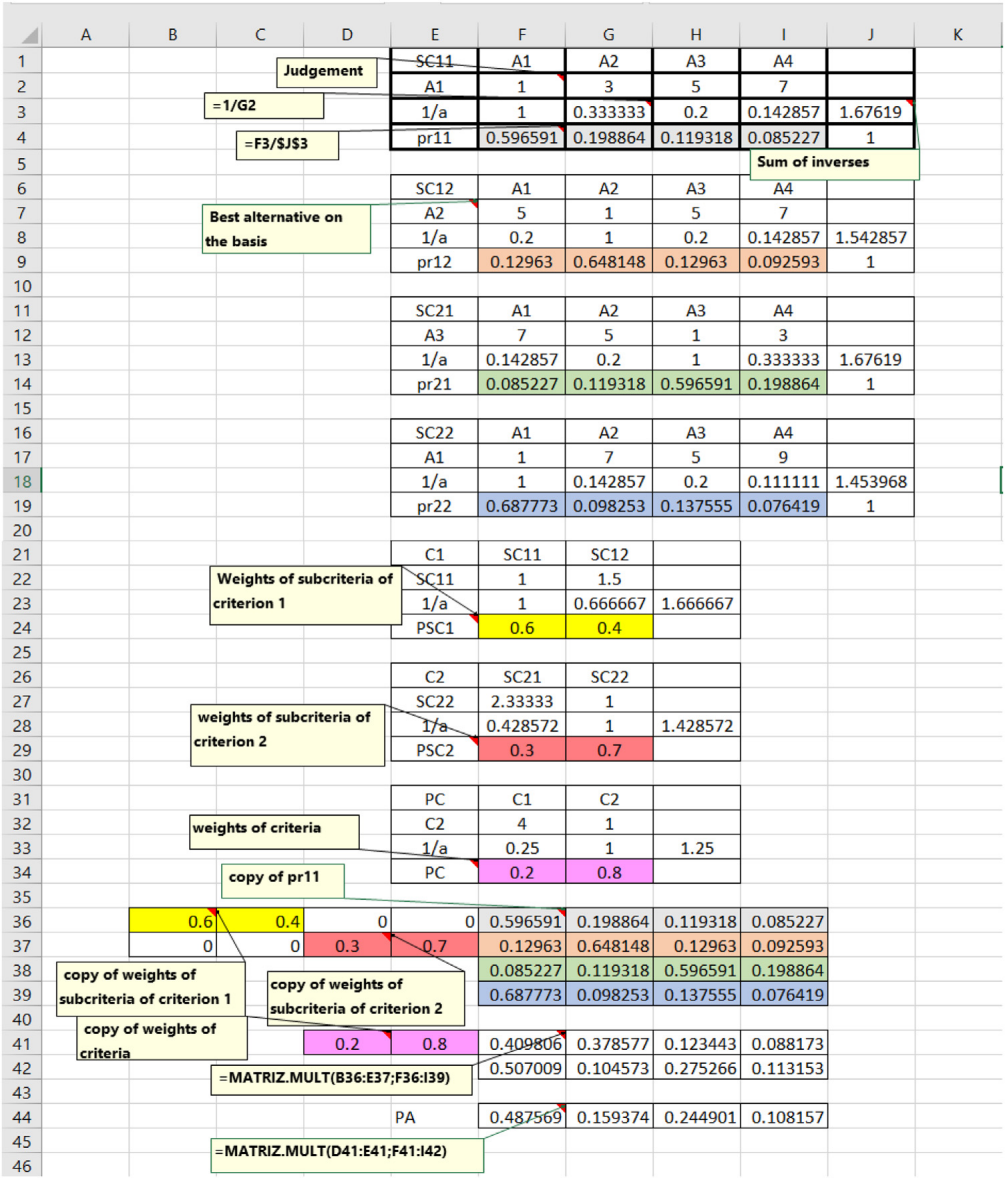
AHP-express process in an Excel spreadsheet.

**Fig. 4:**
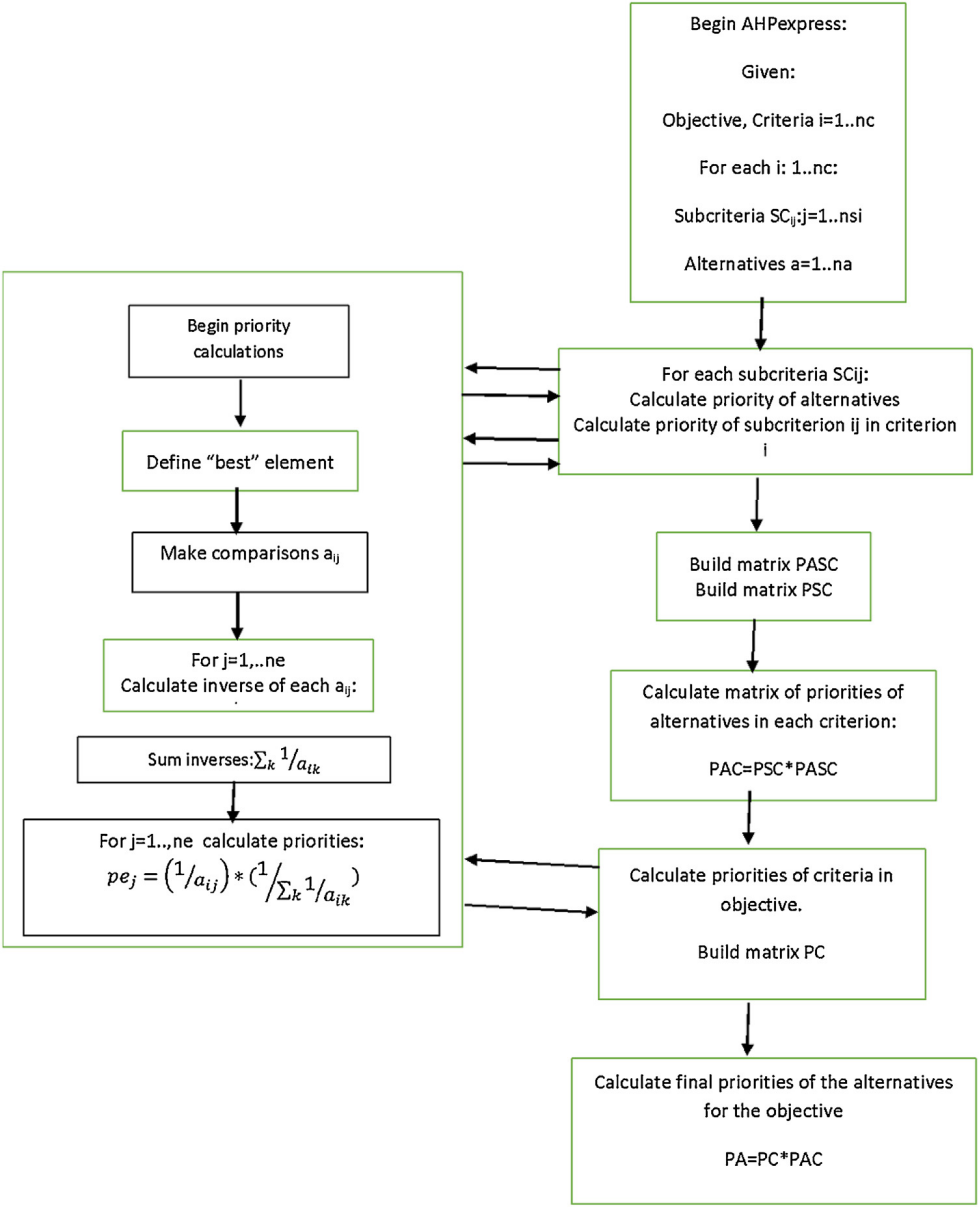
Diagram with steps of Ahp-express for a hierarchy with criteria, subcriteria and alternatives.

The Microsoft Excel worksheet in Fig. 3 shows the entire calculation process. The main formulas are shown in the figure.

First, the priority is calculated for each alternative in each subcriterion SC11, SC12, SC21 and SC22. One then calculates the PSC1 and PSC2 priorities of each subcriterion for each criterion and the priorities of each criterion for the final objective in the PC vector.

The vectors of priorities of each alternative in each subcriterion are sorted and grouped in the MPASC matrix. In turn, the subcriteria vectors are grouped into a partitioned MPSC matrix to match the rows of the MPASC matrix. The product of the MPSC matrix and the MPASC matrix gives the PAC matrix of priorities of the alternatives in each criterion, shown in rows 36–39. The result is premultiplied (rows 41 and 42) by the vector of priorities of the criteria and results in the final PA vector of priorities of each alternative for the final objective, row 44.

This finding indicates that for this decision-making process, the A1 alternative with priority 0.62 is dominant over the others, and the alternative A4 has the second priority, which is well below that of A1.
