# Supplementary material for: AHP-express: A simplified version of the analytical hierarchy process method
Source: MethodsX. 2019 Dec 4;7:100748. doi: 10.1016/j.mex.2019.11.021 (PMC6993013; doi:10.1016/j.mex.2019.11.021)
Supplement: Supplementary file 1 [file mmc1.doc]

**Appendix 1: Approximate methods of calculating the eigenvector and eigenvalue.**

Saaty (2003) considered the eigenvector to be a necessary representation of the vector of priorities extracted from a pairwise comparison matrix between alternatives. In turn, Sekitami and Yamaki (1999) performed a study proving the logic of using the eigenvalue method in the AHP as proposed by Saaty. The following are two methods to obtain an approximation of the eigenvector and the associated eigenvalue to measure the evaluation consistency, as proposed by Saaty.

The method suggested by Winston (1994) to calculate the vector of priorities of an evaluation matrix is ​​shown below.

Take the comparison matrix ***A*** and add up all the elements of each column, as shown in Figure A1.

| Alternative | 1 | . | j | . | n |
| --- | --- | --- | --- | --- | --- |
| 1 |  |  |  |  |  |
| . |  |  |  |  |  |
| i |  |  | aij |  |  |
| . |  |  |  |  |  |
| n |  |  |  |  |  |
| Sum | S1 | . | Sj | . | Sn |

Figure A1: Step 1: establishing the priority estimation vector.

Divide the elements of each column by the sum of the column and calculate the mean in each row. This mean is the priority value of alternative ***i,*** as shown in Figure A2. The priority column shows the estimate of the ***x*** eigenvector associated with the largest eigenvalue of matrix ***A****.*

| Alternative | 1 | . | j | . | n | Priority |
| --- | --- | --- | --- | --- | --- | --- |
| 1 |  |  |  |  |  |  |
| . |  |  |  |  |  |  |
| i |  |  | aij/Sj |  |  | Average i |
| . |  |  |  |  |  |  |
| n |  |  |  |  |  |  |

Figure A2: Step 2: priority estimation.

After obtaining the vector ***x***, the evaluation consistency index can be estimated after estimating the largest eigenvalue ***λ*** associated with matrix ***A*** from the relationship ***Ax=λx***. The consistency index is given by the following formula:

. (A1)

To verify the acceptability of consistency, this value is compared with a table suggested by Saaty, with CI values ​​for matrices of size n, with random evaluation values. Saaty calls these values RI values.

|  | n | 2 | 3 | 4 | 5 | 6 | 7 | 8 | 9 | 10 |
| --- | --- | --- | --- | --- | --- | --- | --- | --- | --- | --- |
|  | RI | 0 | 0.58 | 0.90 | 1.12 | 1.24 | 1.32 | 1.41 | 1.45 | 1.51 |

To perform the consistency test, it is necessary to calculate the consistency ratio, which is performed by dividing the CI of matrix A by the corresponding value of RI. In this case,

|  |  |
| --- | --- |


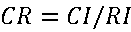
. (A2)

The resulting CR value must be less than 0.1 for the matrix inconsistency to be considered acceptable. Otherwise, the comparison process should be repeated.

Another simplified method proposed by Saaty (2013) is summarized as follows. In matrix A, the value in each row is added to the total value of the elements. The sum of each row is then divided by the total sum to obtain the estimate of the vector of priorities, as shown in Figure 3.

| Alternative | 1 | . | j | . | n | Sum | Priority |
| --- | --- | --- | --- | --- | --- | --- | --- |
| 1 |  |  |  |  |  |  |  |
| . |  |  |  |  |  |  |  |
| i |  |  | aij |  |  | Sumi | Sumi/Tsum |
| . |  |  |  |  |  |  |  |
| n |  |  |  |  |  |  |  |
|  |  |  |  |  |  | Tsum |  |

Figure A3. Illustration of the estimate of the eigenvector according to Saaty.

Then, the estimate of the eigenvalue ***λ***, the consistency index calculation, and the consistency test are performed in the same way as shown above.

**Appendix 2: Deduction of the formula of the simplified method, assuming consistency.**

A2.1: Deduction from Saaty’s approximate method

In this appendix, a simplified procedure is presented to quickly calculate the priorities from the comparison of a single element with the others, assuming evaluation consistency. Note that if consistency is maintained, then one can calculate the evaluation matrix from an evaluation between one alternative i and all the other alternative and construct the complete matrix using the following relation:

, (A3)

where k and j are two alternatives that are compared to i.

3.1. Deduction of the simplified formula from Saaty’s approximate method.

The sum of each row j of the matrix is as follows:

. (A4)

The total sum of the matrix, or the sum of the totals of the rows, is

. (A5)

Then, the evaluation with the priority pr_j_ for the row or alternative j is

. (A6)

By taking the first element as a reference in the matrix (i=1), the resulting value is 1.

Thus, the priority is

. (A7)

The other elements of j are

. (A8)

Then, a general formula for calculating the priority of all elements can be defined when starting from any row i, that is, comparing one element i with all the others:


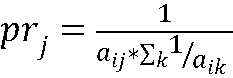
. (A9)

A2.2. Deduction by the calculation of the eigenvalue and eigenvector

In this section, it is proven that the vector of priorities obtained with the formula is the eigenvector that would be obtained from a consistent matrix. First, we determine the eigenvalues ​​of a comparison matrix.

Let the characteristic determinant be a consistent matrix of dimension 3.


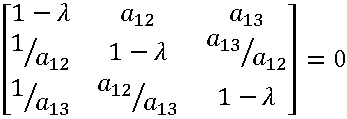
.

By developing this expression, we arrive at the following third-degree equation:


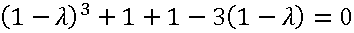
,

which becomes


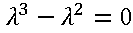
,

with the roots 0, 0 and 3. Therefore, the largest eigenvalue of a consistent matrix is ​​equal to the dimension of the matrix, which in this case is 3.

The eigenvector corresponding to the highest eigenvalue is obtained by the system of equations obtained from


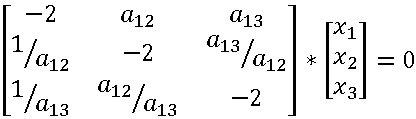
,


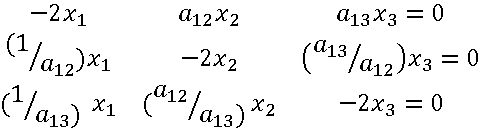
.

From this system, one obtains
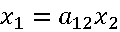
 and
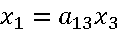


From this result, we would have an eigenvector proportional to


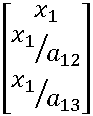
.

The vector of priorities is normalized to sum to 1.

By dividing each element of the matrix by that sum, we have
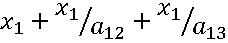
, and we obtain the following vector of priorities:


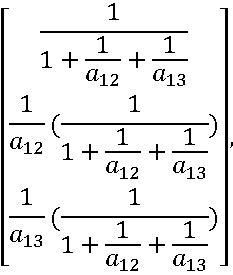


as proposed in the method.

This procedure can be applied to a consistent matrix of any dimension *n*; it produces a maximum eigenvalue=*n* and a corresponding eigenvector with *n* elements that are already normalized to sum to 1:


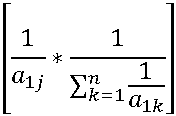


Therefore, the method does not give an approximation of the vector of priorities; instead, it yields the exact value of the vector of priorities, which is the eigenvector corresponding to the highest eigenvalue, normalized to 1.
